# Relation of hypoxia inducible factor 1**α** and 2**α** in operable non-small cell lung cancer to angiogenic/molecular profile of tumours and survival

**DOI:** 10.1054/bjoc.2001.2018

**Published:** 2001-09

**Authors:** A Giatromanolaki, M I Koukourakis, E Sivridis, H Turley, K Talks, F Pezzella, K C Gatter, A L Harris

**Affiliations:** Departments of Pathology and Radiotherapy / Oncology, Democritus University of Thrace, P.O.Box 128, Alexandroupolis, 68100, Greece; Nuffield Department of Clinical Laboratory Sciences, John Radcliffe Hospital, Oxford, OX3 7LJ, UK; Imperial Cancer Research Fund, Molecular Oncology Laboratories, Institute of Molecular Medicine, John Radcliffe Hospital, Oxford, OX3 9DS, UK

**Keywords:** non-small-cell lung cancer, hypoxia inducible factors, angiogenesis, prognosis

## Abstract

Hypoxia inducible factors HIF1α and HIF2α are important proteins involved in the regulation of the transcription of a variety of genes related to erythropoiesis, glycolysis and angiogenesis. Hypoxic stimulation results in rapid increase of the HIF1α and 2α protein levels, as a consequence of a redox-sensitive stabilization. The HIFαs enter the nucleus, heterodimerize with the HIF1β protein, and bind to DNA at the hypoxia response elements (HREs) of target genes. In this study we evaluated the immunohistochemical expression of these proteins in 108 tissue samples from non-small-cell lung cancer (NSCLC) and in normal lung tissues. Both proteins showed a mixed cytoplasmic/nuclear pattern of expression in cancer cells, tumoural vessels and tumour-infiltrating macrophages, as well as in areas of metaplasia, while normal lung components showed negative or very weak cytoplasmic staining. Positive HIF1α and HIF2α expression was noted in 68/108 (62%) and in 54/108 (50%) of cases respectively. Correlation analysis of HIF2α expression with HIF1α expression showed a significant association (*P* < 0.0001, r = 0.44). A strong association of the expression of both proteins with the angiogenic factors VEGF (*P* < 0.004), PD-ECGF (*P* < 0.003) and bFGF (*P* < 0.04) was noted. HIF1α correlated with the expression of bek-bFGF receptor expression (*P* = 0.01), while HIF2α was associated with intense VEGF/KDR-activated vascularization (*P* = 0.002). HIF2α protein was less frequently expressed in cases with a medium microvessel density (MVD); a high rate of expression was noted in cases with both low and high MVD (*P* = 0.006). Analysis of overall survival showed that HIF2α expression was related to poor outcome (*P* = 0.008), even in the group of patients with low MVD (*P* = 0.009). HIF1α expression was marginally associated with poor prognosis (*P* = 0.08). In multivariate analysis HIF2α expression was an independent prognostic indicator (*P* = 0.006, t-ratio 2.7). We conclude that HIF1α and HIF2α overexpression is a common event in NSCLC, which is related to the up-regulation of various angiogenic factors and with poor prognosis. Targeting the HIF pathway may prove of importance in the treatment of NSCLC. © 2001 Cancer Research Campaignhttp://www.bjcancer.com


*British Journal of Cancer* (2001) **85**(6), 881–890
© 2001 Cancer Research Campaign
doi: 10.1054/ bjoc.2001.2018, available online at http://www.idealibrary.com on http://www.bjcancer.com

### Relation of hypoxia inducible factor 1α and 2α in operable non-small cell lung cancer to angiogenic/molecular profile of tumours and survival

**A Giatromanolaki[1], MI Koukourakis[1], E Sivridis[1], H Turley[2], K Talks[2], F Pezzella[2], KC Gatter[2] and AL Harris[3]**

[1]Departments of Pathology and Radiotherapy / Oncology, Democritus University of Thrace, P.O.Box 128, Alexandroupolis 68100, Greece; [2]Nuffield Department of Clinical Laboratory Sciences, John Radcliffe Hospital, Oxford, OX3 7LJ, UK; [3]Imperial Cancer Research Fund, Molecular Oncology Laboratories, Institute of Molecular Medicine, John Radcliffe Hospital, Oxford OX3 9DS, UK

**Summary** Hypoxia inducible factors HIF1α and HIF2α are important proteins involved in the regulation of the transcription of a variety of genes related to erythropoiesis, glycolysis and angiogenesis. Hypoxic stimulation results in rapid increase of the HIF1α and 2α protein levels, as a consequence of a redox-sensitive stabilization. The HIFαs enter the nucleus, heterodimerize with the HIF1β protein, and bind to DNA at the hypoxia response elements (HREs) of target genes. In this study we evaluated the immunohistochemical expression of these proteins in 108 tissue samples from non-small-cell lung cancer (NSCLC) and in normal lung tissues. Both proteins showed a mixed cytoplasmic/nuclear pattern of expression in cancer cells, tumoural vessels and tumour-infiltrating macrophages, as well as in areas of metaplasia, while normal lung components showed negative or very weak cytoplasmic staining. Positive HIF1α and HIF2α expression was noted in 68/108 (62%) and in 54/108 (50%) of cases respectively. Correlation analysis of HIF2α expression with HIF1α expression showed a significant association ($P <$ 0.0001, r = 0.44). A strong association of the expression of both proteins with the angiogenic factors VEGF ($P < 0.004$), PD-ECGF ($P < 0.003$) and bFGF ($P < 0.04$) was noted. HIF1α correlated with the expression of bek-bFGF receptor expression ($P = 0.01$), while HIF2α was associated with intense VEGF/KDR-activated vascularization ($P = 0.002$). HIF2α protein was less frequently expressed in cases with a medium microvessel density (MVD); a high rate of expression was noted in cases with both low and high MVD ($P = 0.006$). Analysis of overall survival showed that HIF2α expression was related to poor outcome ($P = 0.008$), even in the group of patients with low MVD ($P = 0.009$). HIF1α expression was marginally associated with poor prognosis ($P = 0.08$). In multivariate analysis HIF2α expression was an independent prognostic indicator ($P = 0.006$, t-ratio 2.7). We conclude that HIF1α and HIF2α overexpression is a common event in NSCLC, which is related to the up-regulation of various angiogenic factors and with poor prognosis. Targeting the HIF pathway may prove of importance in the treatment of NSCLC. © 2001 Cancer Research Campaign http://www.bjcancer.com

**Keywords**: non-small-cell lung cancer; hypoxia inducible factors; angiogenesis; prognosis

Hypoxia has been recognized as a key factor involved in the regulation of a cascade of physiological responses such as erythropoiesis (Gopfert et al, 1996), glycolysis (Ebert et al, 1996), glucose transport (Tagaki et al, 1998), angiogenesis and vasodilatation (Griffiths et al, 1997; Carmeliet et al, 1998; Faller, 1999). A large group of genes that includes erythropoietin (Goldberg et al, 1988), nitric oxide synthase (Palmer et al, 1998), tyrosine hydroxylase (Norris and Millhorn, 1995), VEGF (Forsythe et al, 1996; Ema et al, 1997), lactate dehydrogenase, haem oxygenase, transferin receptor and others are regulated by hypoxia (Blancher and Harris, 1998). Cells, whether normal or malignant, have the ability to ‘sense’ low oxygen conditions, probably via a haem flavo-oxido-reductase protein or even through hypoxia-stimulated release of reactive oxygen species from mitochondria (Chandel et al, 2000), which activates a signalling pathway for the expression of the hypoxia-regulated genes.

HIF1 is a heterodimer of 2 basic-helix-loop-helix PAS domain proteins; HIF1α (120 kDa protein) and HIF1β (91–94 kDa protein also called as aryl-hydrocarbon-nuclear receptor translocator, ARNT1 and 2) (Semenza et al, 1991; Semenza and Wang 1992). Increased intracellular content of the hypoxia-inducible factor 1 (HIF1) occurs immediately following hypoxia sensing. HIF1β is present constitutively and usually does not change following hypoxic stimulation. In contrast, HIF1α levels are maintained at low levels under normoxic conditions due to continuous degradation via the ubiquitin-dependent proteasome pathway (Huang et al, 1996). Hypoxic stimulation results in rapid increase of the HIF1α protein levels, which is not a result of increased mRNA transcription or translation but rather a result of a redox-sensitive stabilization (Huang et al, 1998). Following HIF1 heterodimerization the complex enters into the nucleus and binds to DNA at the hypoxia response elements (HREs) of target genes.

More recently new molecules, HIF2α, (or endothelial PAS domain protein 1, EPAS1 or HIF1α-like factor, HLF) and HIF3α have been identified with very similar characteristics to HIF1α in dimerization with HIF1β and DNA binding (Ema et al, 1997; Gu et al, 1998). HIF2 regulates, similarly to HIF1, the transcription of hypoxia-regulated genes such as *VEGF* and the endothelial cell-specific receptor tyrosine kinase gene *Tie-2* (Tian et al, 1997). HIF2α was believed to be specifically expressed in endothelial cells and is an important molecule during vasculogenesis (Tian et al, 1997). A recent study, however, showed that HIF2α is also expressed in fibroblasts and in epithelial cells (Wiesner et al, 1998).

*Received 27 March 2001*
*Revised 4 June 2001*
*Accepted 12 June 2001*

*Correspondence to:* AL Harris

In the present study we examined the expression patterns of HIF1α and of HIF2α in normal lung and in a series of non-small-cell lung carcinomas. The expression of HIFs was analysed in comparison with the microvessel density, with the expression of multiple angiogenic factors and receptors as well as with the expression of onco-proteins, previously shown to have a role in lung cancer. The prognostic role of HIF expression was also studied.

### MATERIALS AND METHODS

We examined 108 tumour samples from patients with early operable (T1,2-N0,1-M0 staged) non-small-cell lung cancer (72 squamous and 36 adenocarcinomas). 12 samples from normal lung obtained during thoracic surgery for reasons other than lung cancer were also examined. Histological diagnosis, grading and N-stage was done on haematoxylin-eosin stained sections. 48 patients had T1-stage and 60 T2-stage disease. Node involvement (N1-stage) was present in 34/108 patients. Histological grade 3 was noted in 59/108 and grade 1/2 in 49/108 patients. 84 patients were male and 24 female, their ages ranging from 35 to 74 years (median 63). Survival data (overall survival) were available in 98/108 patients. Patients dying within 60 days after operation were excluded, so as to avoid bias from perioperative death. The follow-up of surviving patients at the time of analysis was 621–2500 days (median 1720 days).

### Assessment of HIF1α and HIF2α protein expression

The HIF1α and HIF2α proteins were detected using the ESEE 122 (IgG1 Mab; dilution 1:20) and the EP190b (IgG1 Mab; neat) as we previously described (Wiesner et al, 1998; Talks et al, 2000). Sections were deparaffinized and peroxidase was quenched with methanol and $H_2O_2$ 3% for 15 minutes. Microwaving for antigen retrieval was used (3 × 4 min). The primary antibodies were applied for 90 minutes. Following washing with TBS, sections were incubated with a secondary anti-rabbit anti-mouse antibody (Kwik Biotinylated Secondary, 0.69A Shandon-Upshaw) for 15 min and washed in TBS. Kwik Streptavidin peroxidase reagent (039A Shandon-Upshaw) was applied for 15 min and sections were again washed in TBS. The colour was developed by 15 min incubation with DAB solution and sections were weakly counterstained with haematoxylin. Breast cancer tissue sections with strong nuclear HIF1α and HIF2α expression were used as positive controls. Normal mouse immunoglobulin-G was substituted for primary antibody as the negative control (same concentration as the test antibody).

The percentage of cells expressing HIF1α or HIF2α in the cytoplasm and in the nuclei was separately assessed. The intensity of the cytoplasmic staining was also scored as absent, weak, moderate and strong.

### Assessment of microvessel density

The JC70 monoclonal antibody (DAKO) recognizing the CD31 pan-endothelial antigen (platelet/endothelial cell adhesion molecule; PECAM-1) was used for microvessel and single endothelial cell staining on 5 μm paraffin-embedded sections. We used the alkaline phosphatase/anti-alkaline phosphatase (APAAP) procedure as previously described (Giatromanolaki et al, 1998). Sections were dewaxed, rehydrated and pre-digested with protease type XXIV for 20 min at 37˚C. JC70 (1:50) was applied at room temperature for 30 min and washed in TBS. Rabbit anti-mouse antibody 1:50 (v/v) was applied for 30 min, followed by application of mouse APAAP complex 1:1 (v/v) for 30 min. After washing in TBS, the last 2 steps were repeated for 10 min each. The colour was developed by 20 min incubation with New Fuchsin solution.

Microvessel counting was used for angiogenesis assessment. For eye appraisal sections were scanned at low power (× 40 and × 100) and afterwards at × 200 field so as to group cases in 3 vascular grade categories (low, medium and high). The areas of the highest vascularization were chosen at low power (× 100) and microvessel counting followed on 3 chosen × 200 fields of the highest density. The microvessel score (MS) was the sum of the vessel counts obtained in these 3 fields. Microvessels adjacent to normal lung were excluded from the appraisal. Vessels with a clearly defined lumen or well defined linear vessel shape but not single endothelial cells were taken into account for microvessel counting.

### Assessment of VEGF and of VEGF/KDR-activated microvessel density

The VEGF expression was assessed with the VG1 Mab (IgG isotype) recognizing the 121, 165 and 189 isoforms of *VEGF* (21). The VEGF/KDR complex was assessed with the 11β5 Mab, an IgMμ isotype produced using the VEGF Hu NH2-terminus as an immunogen (Brekken et al, 1998). Sections were dewaxed, rehydrated and microwaving (4 min × 2) for antigen retrieval was applied. 5 μm paraffin-embedded sections were stained using the alkaline phosphatase/anti-alkaline phosphatase (APAAP) procedure. The primary Abs (1:4 dilution) were applied at room temperature for 1 hour and washed in TBS. Rabbit anti-mouse antibody 1:50 (v/v) was applied for 30 min, followed by application of mouse APAAP complex 1:1 (v/v) for 30 min. After washing in TBS, the last 2 steps were repeated for 10 min each. The colour was developed by 15 min incubation with Fast red solution and sections were weakly counterstained with haematoxylin. Non-specific immunoglobulins were substituted for primary antibody as negative controls (same concentration as the test antibody).

The percentage of cancer cells with cytoplasmic VEGF reactivity was assessed by 2 independent observers at × 200 magnification as previously reported (Giatromanolaki et al, 1998). The VEGF/KDR-positive microvessel density (activated MVD, aMVD) was assessed at the tumoural invading front as previously reported (Koukourakis et al, 2000).

### Assessment of thymidine phosphorylase expression

Thymidine phosphorylase (TP; platelet-derived endothelial cell growth factor, PD-ECGF) expression was assessed with the P-GF.44C monoclonal antibody using the streptavidin-biotin-peroxidase technique as previously described (Koukourakis et al, 1997a). The percentage of cancer cells with strong cytoplasmic/nuclear reactivity was recorded.

### Assessment of bFGF and bFGF-Bek-receptor expression

The cytoplasmic basic Fibroblast Growth Factor (bFGF) and its ‘bek’ receptor (FGFR-2) expression was assessed in cancer cells,

 © 2001 Cancer Research Campaign using the APAAP technique. We used the FGF-2 (147)-G and the Bek(C-17)-G MAbs respectively (Santa Cruz Biotechnology) as previously reported (Giatromanolaki et al, 2000). The percentage of cancer cells with positive cytoplasmic reactivity was recorded.

### Assessment of other immunohistochemical variables

Proliferative index was assessed with the monoclonal antibody Ki67. Frozen material was taken from 2 separate areas of the tumour and the Ki67 assessment was based on the average value. Three groups were considered based on the percent of stained nuclei: 0–10% = low proliferative index, 10–40% = medium and > 40% = high (Tungekar et al, 1991).

The bcl-2 cytoplasmic protein expression and the p53 protein nuclear accumulation was assessed with the clone 100 (Dako) and the CM11(Dako) MoAbs respectively, as previously described (Giatromanolaki et al, 1998).

The EGFR, c-erbB-2 and episialin MUC-1 expression was also assessed (data not shown).

### Assessment of necrosis

The percentage of optical fields ( × 250) with necrosis was recorded by 3 observers separately. Necrotic areas in more than 50% of the examined fields (mean value of the score given by the observers) was scored as extensive and, in less than 50%, as limited.

### Statistical analysis

Statistical analysis and graphic presentation were performed using the GraphPad Prism 2.01 package (GraphPad, San Diego CA, www.graphpad.com). The Fisher's exact test, the chi-square *t*-test or the unpaired two-tailed *t*-test was used for testing relationships between categorical variables as appropriate. Linear regression analysis was used to assess correlation between continues variables. Survival curves were plotted using the method of Kaplan–Meier, and the log-rank test was used to determine statistical differences between life tables. A Cox proportional hazard model was used to assess the effects of patient and tumour variables on overall survival. A $P$ value ≤ 0.05 was considered significant.

### RESULTS

### Immunostaining of normal lung and cancer

Both HIF1α and HIF2α showed a similar pattern of normal tissue staining. Samples from normal lung and areas distal to the tumour showed a weak or negative cytoplasmic reactivity of the bronceal epithelium, while alveolar epithelium and stroma were negative (Figure 1A). Normal bronchial and alveolar epithelium proximal to the tumour showed weak or even intense cytoplasmic staining. Alveolar macrophages and plasma cells in the normal lung were negative (Figure 1E), while tumour-infiltrating macrophages (CD68 positive cells identified with immunohistochemistry in parallel sections) showed intense cytoplasmic and nuclear reactivity (Figure 1F). Chondrocytes persistently showed a pure and strong nuclear reactivity (Figure 1A), which renders this finding an internal marker of positive staining. Normal vasculature was negative, while a strong cytoplasmic and/or nuclear staining of the endothelium was noted within the tumoural tissue and the invading front (Figure 1G). Areas of squamous metaplasia showed strong mixed cytoplasmic/nuclear reactivity (Figure 2A).

The expression of both HIF1α and HIF2α in cancer cells, both in adenocarcinomas and squamous tumours, was mixed cytoplasmic and nuclear (Figures 1C,D and 2B,C) and the percentage of stained cells was largely varying among cases. The intensity of staining varied among cases. Absent or weak staining was considered as negative, while moderate or strong as positive. Cytoplasmic staining of the stromal fibroblasts also varied among samples.

### Scoring of HIF expression in cancer cells

HIF1α and HIF2α expression was both cytoplasmic and nuclear. Cytoplasmic staining was scored as absent, weak, moderate and strong. Nuclear expression, when present, was accompanied with moderate/strong cytoplasmic reactivity, although pure nuclear expression was occasionally noted. The extent of staining also varied among tumours. The percentage of cancer cells with HIF1α moderate/strong cytoplasmic (and/or nuclear) reactivity ranged from 0% to 90% (median 80%; mean 63%). The percentage of HIF2α-positive cancer cells with moderate/strong cytoplasmic (and/or nuclear) expression ranged from 0% to 90% (median 45%; mean 43%).

Tumours were scored in a 3-scale system according to the intensity and extent of staining: score 1, tumours with absent or weak cytoplasmic reactivity and no nuclear reactivity; score 2, tumours with moderate/strong cytoplasmic reactivity in a percentage of cancer cells lower than the mean value and no nuclear reactivity; score 3, tumours with moderate/strong cytoplasmic reactivity in a percentage of cancer cells higher than the mean value; score 4, tumours with a clear nuclear reactivity (with or without cytoplasmic reactivity regardless of the intensity). Tumours with score 1 and 2 were grouped as bearing low HIF reactivity, while tumours with score 2 and 3 as bearing high HIF reactivity. Using these criteria, 40 cases had low and 68 high HIFα reactivity. 54 cases had low and 44 had high HIFα reactivity.

### Correlation between HIF1α and HIF2α

Correlation analysis of HIF2α expression with HIF1α expression showed a significant association ($P < 0.0001$, r = 0.44). Out of 68 cases with high HIF1α expression 43 (63%) also had high HIF2α expression, while 11/29 (37%) cases with low HIF1α expression had high HIF2α expression ($P = 0.0006$; Fisher's exact *t*-test).

### HIF1α/2α and histopathological variables

HIF2α expression was significantly more frequent in squamous cell carcinomas than in adenocarcinomas (30/72 vs. 24/36; $P = 0.02$), while HIF α was not related to histology. Although HIF1α expression was more frequent in T2 than in T1 stage the difference was not significant (42/60 (70%) vs. 26/48 (54%); $P = 0.11$). High Ki67 proliferation index was not related to either HIF1α and HIF2α expression ($P > 0.81$). There was no association of HIF1α and HIF2α expression with N-stage, histological grade, or the extent of necrosis (data not shown)

### HIF1α/2α and angiogenic profile of tumours

Using the 33rd and 66th percentile as cut-off point we grouped our cases in 3 categories of low, medium and high angiogenic

© 2001 Cancer Research Campaign

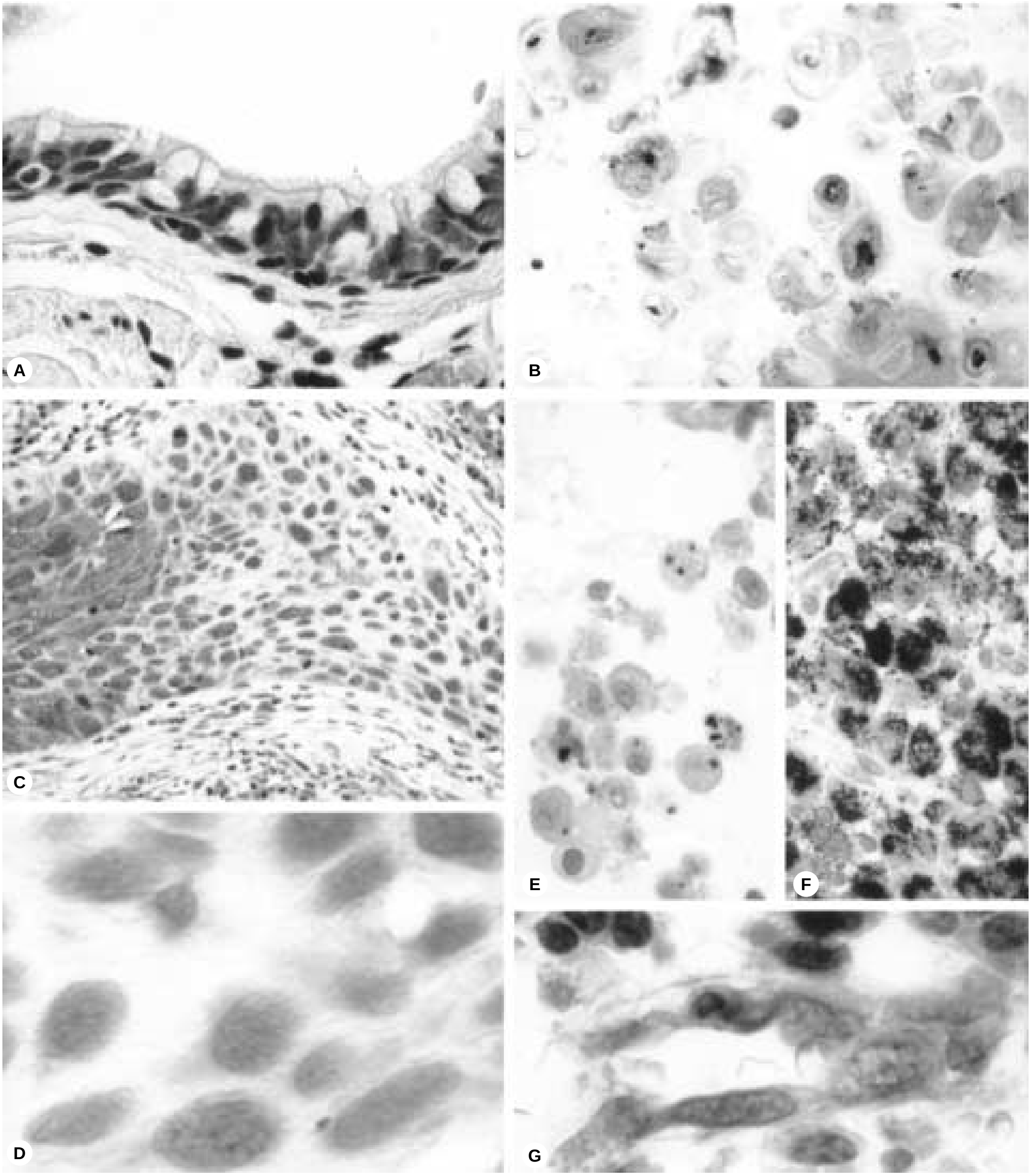

**Figure 1** Expression of HIF1α in normal and neoplastic lung. (**A**) Normal bronchial epithelium does not express HIF1α (× 200); (**B**) Nuclear staining of chondrocytes (× 400); (**C**) Nuclear and mixed cytoplasmic/nuclear HIF1α staining of squamous cell lung cancer (× 200); (**D**) Absence of HIF1α staining in alveolar macrophages (× 400); (**E**) Intense HIF1α staining of tumour-infiltrating macrophages (× 400); (**F**) Nuclear HIF1α staining of squamous cancer cells (× 400); (**G**) Nuclear/cytoplasmic HIF1α staining of the endothelium (× 400)

factor and receptor expression. For VEGF, low, medium and high expression refers to 0–30%, 31–69% and 70–100% of cells with positive reactivity. For TP, low, medium and high expression refer to 0–10%, 11–49% and 50–100% of cells with strong TP reactivity. For bFGF, low, medium and high expression refer to 0–59%, 60–79% and 80–100% of cells stained. For bFGF-bek-receptor the percentage of stained cells used to define low, medium and high reactivity were 0–9%, 10–20% and > 20% respectively. Cases with < 4, 4–10 and > 10 VEGF/KDR-positive vessels in the tumour-invading front (points that correspond to the 33rd and 66th percentile) were grouped as bearing low, medium and high activated MVD respectively. Similarly, the 33rd and 66th percentile of

 *© 2001 Cancer Research Campaign*

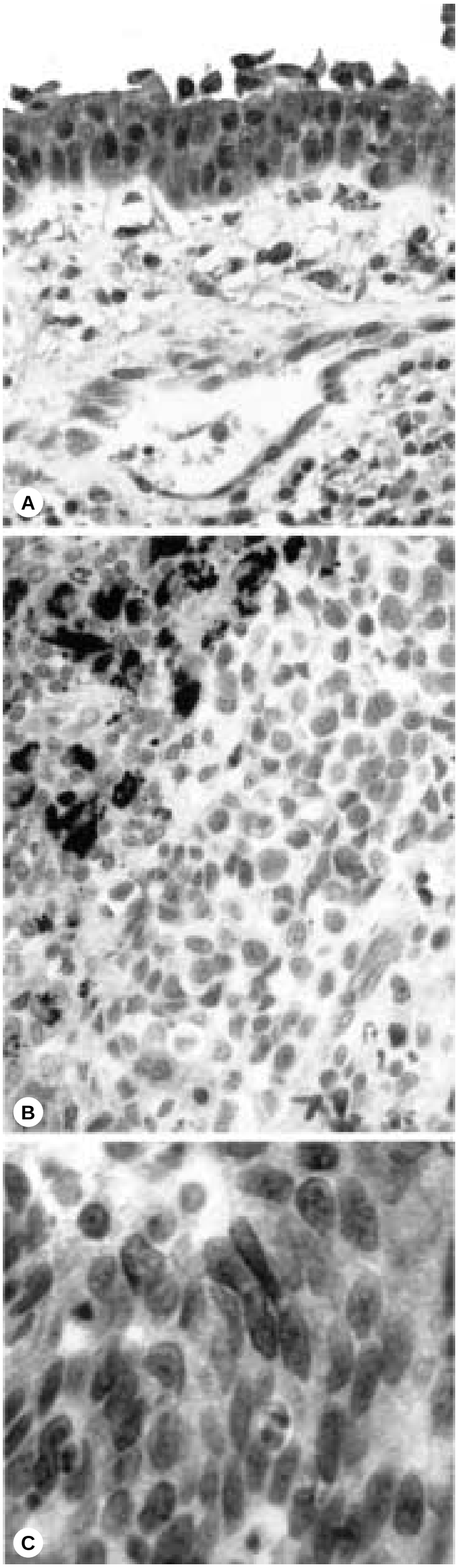

**Figure 2** Expression of HIF2α in metaplasia and neoplastic lung. (**A**) Intense nuclear and cytoplasmic HIF2α expression in lung metaplasia (× 200); (**B**) Nuclear expression of HIF2α in squamous cell lung cancer and tumour-infiltrating macrophages (× 200); (**C**) Nuclear HIF2α expression in squamous cell lung cancer (× 400)

MVD was used to define low (< 32 vessels), medium (33–70 vessels) and high (> 70 vessels) MVD groups.

Table 1 shows the association of HIF1α and HIF2α expression with microvessel density and the expression of angiogenic factors and angiogenic factor receptors. A significant association of both HIF1α and HIF2α with all 3 angiogenic factors (VEGF, PD-ECGF and bFGF) examined was noted. 88% and 70% of cases with high VEGF expression had high HIF1α and HIF2α expression, respectively, vs. 48% (HIF1α) and 38% (HIF2α) of cases with low/medium VEGF expression ($P = 0.0002$; Fisher's exact *t*-test). Similar differences were noted for PD-ECGF (thymidine phosphorylase) and bFGF ($P = 0.0004$ to 0.006). High bFGF-bek-receptor (bFGFR) expression was associated with high HIF1α expression ($P = 0.007$) but not with HIF2α. On the contrary, HIF2α expression was strongly associated with the VEGF/KDR-activated microvessel density in the invading tumour front ($P = 0.0009$), while HIF1α was not. Combining HIF1α and HIF2α expression (low/low vs. all others), a very strong association of HIF overexpression with VEGF ($P < 0.0001$) and with TP ($P = 0.0004$) was noted (data not shown).

Cases with high MVD were more frequently of high HIF1α and HIF2α expression as compared to the group of low/medium MVD, but the difference did not reach significance ($P = 0.18$ and 0.08 for HIF1α and HIF2α, respectively). Of interest, the group of patients with medium MVD was strongly associated with low HIF2α expression, while HIF2α expression was similarly high in cases with low and high MVD ($P = 0.01$ and 0.008, respectively).

A continuous variable analysis revealed that the mean percentage of HIF1α-positive cells (cells with moderate/strong cytoplasmic reactivity or nuclear expression) in cases with low/medium MVD was significantly lower than in cases with high MVD (71 ± 15 vs. 60 ± 26; $P = 0.03$; Figure 3a). Similarly, cases with low/medium

**Table 1** Correlation of HIF1α and HIF2α expression with angiogenic factor expression and microvessel density in non-small-cell lung cancer

| Parameter | HIF1α | | | HIF2α | | |
|---|---|---|---|---|---|---|
| | Low | High | *P* value | Low | High | *P* value |
| VEGF | | | | | | |
| low | 19 | 14 | | 22 | 11 | |
| medium | 16 | 19 | 0.0002 | 20 | 15 | 0.004 |
| high | 5 | 35 | | 12 | 28 | |
| PD-ECGF | | | | | | |
| low | 17 | 22 | | 23 | 16 | |
| medium | 17 | 16 | 0.006 | 22 | 12 | 0.003 |
| high | 6 | 30 | | 10 | 26 | |
| bFGF | | | | | | |
| low | 26 | 18 | | 28 | 16 | |
| medium | 7 | 24 | 0.0004 | 14 | 17 | 0.04 |
| high | 7 | 26 | | 1 | 2 | |
| VEGF/KDR | | | | | | |
| low | 18 | 22 | | 27 | 13 | |
| medium | 12 | 23 | 0.39 | 18 | 17 | 0.002 |
| high | 10 | 23 | | 9 | 24 | |
| bFGFR | | | | | | |
| low | 21 | 22 | | 25 | 18 | |
| medium | 13 | 18 | 0.01 | 16 | 15 | 0.21 |
| high | 6 | 28 | | 13 | 21 | |
| MVD | | | | | | |
| low | 14 | 21 | | 14 | 21 | |
| medium | 18 | 24 | 0.29 | 29 | 13 | 0.006 |
| high | 8 | 23 | | 11 | 20 | |

© 2001 Cancer Research Campaign

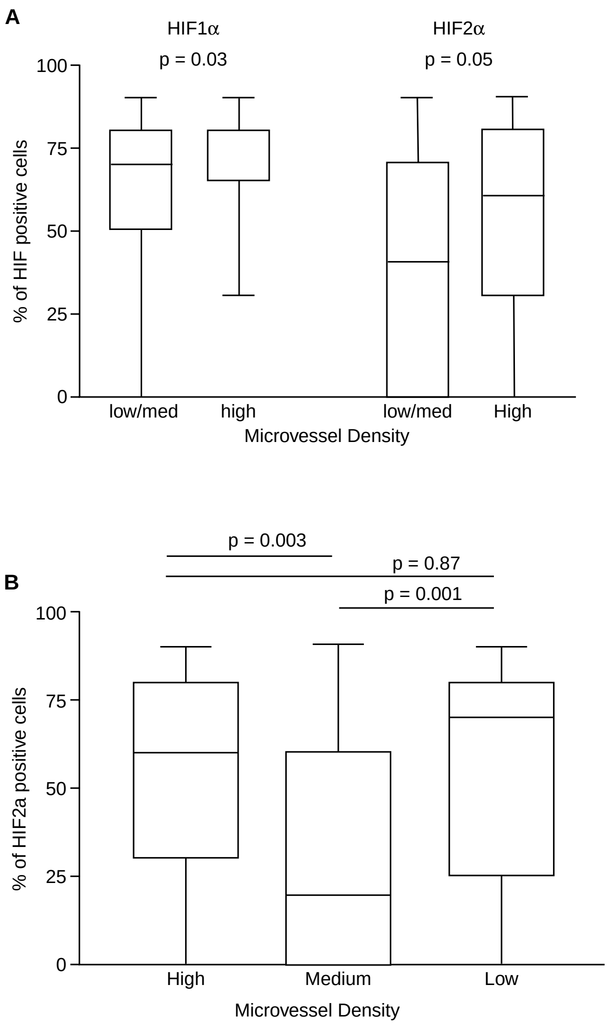

**Figure 3** Percentage of cells with HIF1α and 2α expression in 2 different microvessel density groups (low/medium vs. high; **A**). Percentage of cells with HIF2α expression in 3 different microvessel density groups (low vs. medium vs. high; **B**). Line in the boxes show the median value, the box edges the standard deviation and the bars the range

**Table 2** Correlation of HIF1α and HIF2α expression with cytoplasmic bcl-2 protein expression and p53 protein nuclear accumulation

| **Parameter** | **HIF1α** | | | **HIF2α** | | |
|---|---|---|---|---|---|---|
| | **Low** | **High** | ***P* value** | **Low** | **High** | ***P* value** |
| bcl-2 | | | | | | |
| neg | 28 | 60 | 0.02 | 38 | 50 | 0.005 |
| pos | 12 | 8 | | 16 | 4 | |
| p53 | | | | | | |
| neg | 25 | 28 | 0.04 | 27 | 26 | 0.84 |
| pos | 15 | 40 | | 27 | 28 | |

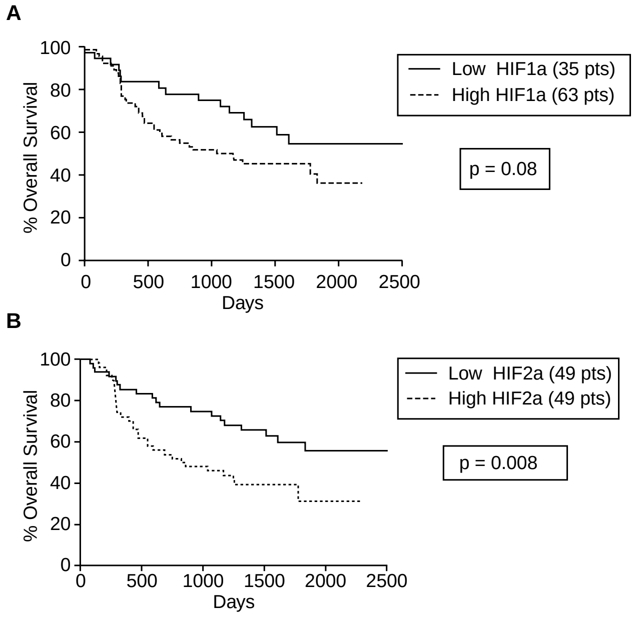

**Figure 4** Kaplan–Meier survival curves of 98 patients with non-small-cell lung cancer stratified for HIF1α (**A**) and HIF2α (**B**) expression (high vs. low)

MVD had a lower percentage of HIF2α-positive cells compared to cases with high MVD (52 ± 26 vs. 39 ± 32; *P* = 0.05; Figure 3a) Further analysis, however, revealed a U-like shaped association of HIF2α expression with MVD is shown in Figure 3b. Cases with high and low MVD had a significantly higher percentage of cells expressing HIF2α as compared to cases with medium MVD.

### HIF1α/2α and other molecular parameters

A significant inverse association of bcl-2 expression with both HIF1α and HIF2α was noted (*P* = 0.02 and 0.005, respectively). p53 nuclear accumulation was significantly more frequent in the HIF1α expressing cases (*P* = 0.04), (Table 2).

No association of HIF1α and HIF2α with EGFR, c-erb B-2 or episialine (MUC1) expression was noted (data not shown). A significant association of HIF2α expression with c-erbB-2 expression was noted in poorly vascularized cases (*P* = 0.04; data not shown).

### HIF1α/2α and survival

Univariate analysis showed that HIF2α expression was significantly associated with poor prognosis (*P* = 0.008), while HIF1α showed a marginal association (*P* = 0.08). Figures 4A,B show the Kaplan–Meier survival curves plotted for HIF1α and HIF2α expression. The 5 year survival of patients with high HIF2α expression was 32% vs. 58% of patients with low HIF2a expression. T,N-stage and MVD were also significantly associated with survival (*P* = 0.006, 0.007 and 0.005, respectively).

We further analysed the survival of patients with low/medium MVD according to the HIF1α and 2α expression (Figure 5A,B). A significantly worse prognosis of patients with low/medium MVD but high HIF2α expression was noted (*P* = 0.04). This association was even stronger in patients with low MVD expressing HIF2α (*P* = 0.009; Figure 5c).

Table 3 shows the results obtained from multivariate analysis of death events in different models including HIFs and the parameters with important prognostic significance in univariate T-stage, N-stage and microvessel density (MVD). Due to the close association of HIF1α and HIF2α with each other and with MVD, as well as due to the close association of N-stage with MVD, only T-stage had an independent prognostic significance in the multivariate model that comprised all the examined parameters. In a multivariate model including T-stage, N-stage, MVD and HIF2α expression we observed that HIF2α had an independent prognostic meaning (*P* = 0.005, t-ratio = 2.0). T-stage was also an independent

 *© 2001 Cancer Research Campaign*

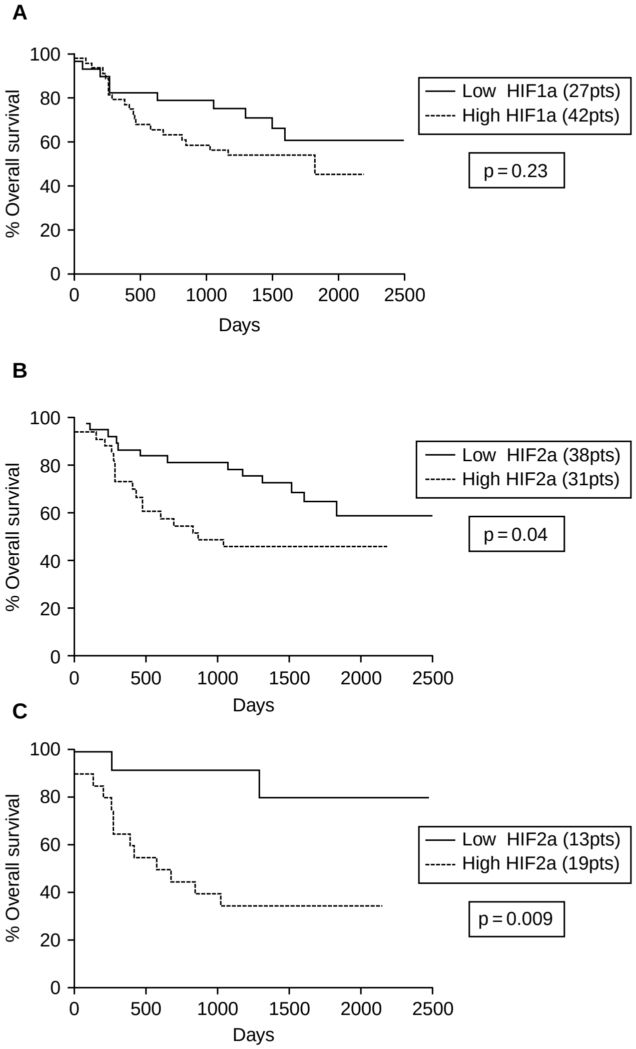

**Figure 5** Kaplan–Meier survival curves in the low/medium microvessel density (MVD) group of patients (69 patients) stratified for HIF1α (**A**) and HIF2α (**B**) expression (positive vs. negative). In (**C**) stratification for HIF2α has been performed in the low MVD group of patients (32 patients)

**Table 3** Multivariate analysis of death events

| Parameter | *P* value/t-ratio | | | | |
|---|---|---|---|---|---|
| | Model 1 | Model 2 | Model 3 | Model 4 | Model 5 |
| HIF1a | 0.57/0.55 | 0.20/1.26 | – | 0.14/1.46 | – |
| HIF2a | 0.11/1.60 | – | 0.04/2.01 | – | 0.03/2.20 |
| T-stage | 0.009/2.65 | 0.01/2.43 | 0.006/2.78 | 0.01/ 2.48 | 0.005/2.86 |
| N-stage | 0.12/1.56 | 0.08/1.75 | 0.13/1.51 | 0.007/2.75 | 0.01/2.39 |
| MVD | 0.13/1.49 | 0.10/1.64 | 0.12/1.55 | – | – |

prognostic variable ($P$ = 0.006, t-ratio = 2.7). N-stage and MVD did not reach significance ($P$ = 0.13 and 0.12) probably because of the strong association of the 2 variables with each other. HIF1α was not revealed as an independent prognostic factor.

### DISCUSSION

Once transformation has occurred, unrestrained cancer cell growth without a parallel formation of vessels leads to the establishment of intratumoral hypoxic conditions. Cells found 100–200 μm away from blood vessels are already hypoxic (Gatenby et al, 1988), which suggests that hypoxic conditions will have been established in a cluster of cells of about 0.5 mm diameter. Activation of the HIF1 pathway may therefore be expected to occur at a very early step of tumour progression. Indeed, HIF1 expression was found to be expressed in in situ carcinomas and also in premalignant conditions (Zhong et al, 1999). In our study we found areas of bronchial metaplasia which exhibited strong staining of both nuclei and cytoplasm. This may be due to early hypoxia, but other mechanisms such as the effects of cytokines e.g. TNFα or growth factors may be responsible. Since HIF1 and HIF2 stabilization up-regulates the expression of angiogenic and glycolytic pathways to restore oxygen homeostasis, HIFs may have an important role for the survival and growth of cancer. Indeed, recent experimental studies confirm that HIFs modulate gene expression resulting in increased angiogenesis and tumoural growth (Jiang et al, 1997; Maxwell et al, 1997).

The significance of HIFs expression in human tumours remains largely unexplored as monoclonal antibodies available for immunohistochemistry have been only recently developed. Zhong et al recently reported on the expression of HIF1α in a panel of normal human tissues and benign or malignant tumours and first showed the expression of the molecule in a good percentage of human carcinomas (Zhong et al, 1999). However, studies on the association of HIF1α and 2α expression with angiogenic factors and receptors, with microvessel density or with other molecular markers or with prognosis of human carcinomas are few.

In a study in brain tumours, Zagzag et al observed that HIF1α and HIF1β expression is expressed around the areas of necrosis and in the invading front of brain tumours (Zagzag et al, 2000). These patterns show that, at least in part, HIF1α expression is a result of differences in tissue oxygenation. In contrast, haemangioblastomas presented a homogeneous expression of HIF1α, suggesting an oncogenic reason for its up-regulation in this tumour type.

Using 2 novel antibodies for HIF1α and HIF2α (Talks et al, 2000), we focused on lung cancer. Expression of HIF1α and 2α by cancer cells was mixed nuclear/cytoplasmic, which is in accordance with the staining patterns reported by Zhong et al (1999). Although it would be assumed that nuclear HIF is the active form, clearly it is synthesized in the cytoplasm and also degraded in the cytoplasm. There may be redistribution while collecting tissues, which would be difficult to control but the overall expression indicates up-regulation of the pathway selectively in cancer. Analysis based on pure nuclear expression showed very marginal or no statistical association with other molecular factors or prognosis, showing that strong cytoplasmic HIF expression, which is a tumour-specific finding, better reflects the HIF up-regulated pathway in paraffin-embedded material. This suggestion is in general accordance with the scoring system proposed by Zhong et al (1999). HIF1α was more frequently overexpressed in lung cancer than HIF2α. Although both molecules were more frequently noted in larger tumours, we found no association of HIF-positive tumours with high Ki67 proliferation index. A strong association of high HIF1α and HIF2α expression with the expression of vascular endothelial growth factor (VEGF), thymidine phosphorylase (TP) and basic fibroblast growth factor (bFGF) was confirmed. Bek bFGF-receptor expression was significantly overexpressed in HIF1α-overexpressing cases, while activated VEGF/KDR angiogenesis was strongly related to HIF2α expression by cancer cells.

© 2001 Cancer Research Campaign 

There was a strong association of HIF expression with multiple angiogenic factors and receptors so a higher MVD was expected in HIF-overexpressing cases. This was the case for HIF2α which was associated with up-regulation of VEGF and its receptor but not for HIF1α where the association was weaker for VEGF and not found for its receptor. For HIF1α there was an association with up-regulation of bFGF and its receptor in contrast to HIF2α. These results will need to be confirmed but do suggest that each HIF may regulate some pathways more than others and may explain the existence of 2 such similar factors.

Although the chi-squared test shows a significant association of HIF2α with MVD ($P$ = 0.006), closer inspection showed a more complicated relationship. HIF2α was higher in cases with high or low MVD but was significantly lower in cases with a medium degree of vascularization. This U-like-shaped association of HIF2α with MVD is in accordance with previous studies of ours where not all VEGF-, TP- or bFGF-expressing tumours show an increase in angiogenesis (Koukourakis et al, 1997b, 2000; Giatromanolaki et al, 1998, 2000). We suggest that although hypoxia triggers the expression of a cascade of angiogenic factors through HIF stabilization, the process of angiogenesis is subject to other modulators. Strong expression of endogenous inhibitors of angiogenesis produced by cancer or stromal cells may therefore counter the effectiveness of the angiogenic cascade released by HIFs (Dong et al, 1997; Koukourakis et al, 1998), while a moderate vascularization may be enough for the restoration of oxygenation that may bring the HIF levels close to the normal level. HIF levels are normalized within minutes after the restoration of oxygenation (Wiesner et al, 1998). The presistent HIF expression in a group of highly angiogenic tumours may show an aggressive phenotype with high oxygen consumption as a result of the transformation itself and not of the hypoxic environment. For example, VHL mutations (Maxwell et al, 1999), activation of HIFs by MAP kinases (Mazure et al, 1999) or the IGF1 pathway (Feldser et al, 1999; Zundel et al, 2000) may be involved in the persistent HIF induction despite the restoration of oxygenated conditions.

Survival analysis showed that HIF2α expression was significantly associated with worse prognosis, which was independent of the MVD. Indeed, stratification of poorly vascularized tumours according to HIF2α expression showed a poorer prognosis of patients with HIF2α overexpression. Patients bearing tumours with low MVD and high HIF1α expression also did worse, but the difference was not significant. These findings show that HIFs may have an important role in tumour progression distinct from angiogenesis. The activation of the glycolytic pathway and increased glucose transport may give a survival and growth advantage to cancer cells growing in hypoxic conditions. A significantly poorer prognosis of patients with early stage HIF1α positive cervical cancer has been also reported by Birner et al (2000), while a putative role of HIF1α in defining poor response to radiotherapy and poor outcome has been reported in a more recent study (Aebersold et al, 2001). In contrast to these studies, Volm et al (2001) found a significant association of HIF1α with a better survival in non-small-cell lung cancer. Although difficult to explain this later observation, an eventual U-like association of HIFs with angiogenesis (intense expression in tumours with very low and with very high angiogenesis) noted in our study shows that the prognostic role of HIFs in human malignancies may be more complicated.

Although, Zhong et al found a significant association of HIF1α expression with Ki67 proliferation index (Zhong et al, 1999), we could not confirm this finding in the present study. Studies on apoptotic index may be useful in identifying a survival advantage conferred by HIFs. We also assessed whether HIF expression was associated with activated migration pathways such as c-erbB-2, EGFR and MUC1/episialin expression. Although such an association was not confirmed overall, analysis in the group of patients with poor vascularization showed a significant association of HIF2α expression with c-erbB-2 overexpression ($P$ = 0.04, data not shown). C-erbB-2 is involved in epithelial cell migration and hypoxia has been shown to induce keratinocyte motility (de-Porter et al, 1994; O'Toole et al, 1997). Whether HIF expression activates migration-related pathways is unknown, but the observed association of HIF2α expression with c-erbB-2 supports our previous findings that c-erbB-2 defines a group of poorly vascularized tumours associated with poor prognosis (Giatromanolaki et al, 1996). A similar association (although of borderline significance, between c-erbB-2 and HIF1a expression has been recently reported in breast cancer (Boss et al, 2001).

Comparative analysis of bcl-2 and HIF expression showed a significant inverse association of both HIF1α and of HIF2α with bcl-2 expression. A similar association has been reported by Zhong et al (1999). In previous studies we showed that bcl-2 expression cancer is associated with poor vascularization in lung and endometrial cancer (Koukourakis et al, 1997; Giatromanolaki et al, 1999) and also in breast cancer (unpublished data). We also reported an inverse association of bcl-2 expression with both VEGF and TP expression (Koukourakis et al, 2000). Indeed, in this study only 3/18 bcl-2-positive tumours had high MVD. All these 3 cases had high HIF expression ($P$ = 0.06; data not shown). The inverse association of bcl2 with HIF stabilization may be because such tumours are characterized by a less aggressive growth, which explains the excellent survival reported in patients with bcl-2-overexpressing lung carcinomas (Koukourakis et al, 1997, 2000). There may therefore be much less oxygen consumption and less metabolically driven hypoxia and, therefore, less induction of HIF. The marginal direct association of HIF1α with p53 nuclear accumulation observed in this study and in the study by Zhong et al (1999) may be due to co-induction of both by hypoxia or stabilizing interactions of both proteins.

We conclude that HIF1α and 2α expression is a common event in lung cancer, which occurs early in the development of the disease as shown by the strong expression in areas of metaplasia. The HIF pathway may therefore be a useful marker in assessing risk of malignancy and will be of interest to assess how this relates to progression and invasion. The HIF transcription pathway may also be a target for cancer prevention. Strong expression of both HIF1α and 2α was related to up-regulation of multiple angiogenic factors and overexpression of angiogenic receptors by cancer cells and the endothelium. HIF2α overexpression showed a strong association with poor survival, even in poorly vascularized cases. Therefore, HIFs confer aggressive tumour behaviour through both angiogenic-dependent and -independent pathways. Targeting the HIF pathway may prove of importance in the treatment of non-small-cell lung cancer.

### REFERENCES

Aebersold DM, Burri P, Beer KT, Laissue J, Djonov V, Greiner RH and Semenza GL (2001) Expression of hypoxia-inducible factor-1α: a novel predictive and prognostic parameter in the radiotherapy of orop haryngeal cancer. *Cancer Res* **61**: 2911–2916

 *© 2001 Cancer Research Campaign*

Birner P, Schindl M, Obermair A, Plank C, Breitenecker G and Oberhuber G (2000) Overexpression of hypoxia-inducible factor 1α is a marker for an unfavorable prognosis in early-stage invasive cervical cancer. *Cancer Res* **60**: 4693–4696

Blancher C and Harris AL (1998) The molecular basis of the hypoxia response pathway: tumour hypoxia as a therapy target. *Cancer Metast Rev* **17**: 187–194

Bos R, Zhong H, Hanrahan CF, Mommers EC, Semenza GL, Pinedo HM, Abeloff MD, Simons JW, van Diest PJ and van der Wall E (2001) Levels of hypoxia-inducible factor-1α during breast carcinogenesis. *J Natl Cancer Inst* **93**: 309–314

Brekken RA, Huang X, King SW and Thorpe PE (1998) Vascular endothelial growth factor as a marker of tumor endothelium. *Cancer Res* **58**: 1952–1959

Carmeliet P, Dor Y, Herbert JM, Fukumura D, Brusselmans K, Dewerchin M, Neeman M, Bono F, Abramovitch R, Maxwell P, Koch CJ, Ratcliffe P, Moons L, Jain RK, Collen D, Keshert E and Keshet E (1998) Role of HIF-1α in hypoxia-mediated apoptosis, cell proliferation and tumour angiogenesis. *Nature* **394**: 485–490

Chandel NS, McClintock DS, Feliciano CE, Wood TM, Melendez JA, Rodriguez AM and Schumacker PT (2000) Reactive oxygen species generated at mitochondrial complex II stabilize HIF-1α during hypoxia: a mechanism of $O_2$ sensing. *J Biol Chem* May 31

de-Porter CR, Eeckhout I, Schelfhout AM, Geerts ML and Roels HJ (1994) Keratinocyte induced chemotaxis in the pathogenesis of Paget’s disease of the breast. *Histopathology* **24**: 349–356

Dong Z, Kumar R, Yang X and Fidler IJ (1997) Macrophage-derived metalloelastase is responsible for the generation of angiostatin in Lewis lung carcinoma. *Cell* **88**: 801–810

Ebert BL, Gleadle JM, O’Rourke JF, Bartlett SM, Poulton J and Ratcliffe PJ (1996) Isoenzyme specific regulation of genes involved in energy metabolism by hypoxia: similarities with the regulation of erythropoietin. *J Biochem* **313**: 809–814

Ema M, Taya S, Yokotani N, Sogawa K, Matsuda Y and Fujii-Kuriyama Y (1997) A novel bHLH-PAS factor with close sequence similarity to hypoxia-inducible factor 1α regulates the VEGF expression and is potentially involved in lung and vascular development. *Proc Natl Acad Sci USA* **94**: 4273–4278

Faller DV (1999) Endothelial cell responses to hypoxic stress. *Clin Exp Pharmacol Physiol* **26**: 74–84

Feldser D, Agani F, Iyer NV, Pak B, Ferreira G and Semenza GL (1999) Reciprocal positive regulation of hypoxia-inducible factor 1α and insulin-like growth factor 2. *Cancer Res* **59**: 3915–3918

Forsythe JA, Jiang BH, Iyer NV, Agani F, Leung SW, Koos RD and Semenza GL (1996) Activation of vascular endothelial growth factor gene transcription by hypoxia-inducible factor 1. *Mol Cell Biol* **16**: 4604–4013

Gatenby RA, Kessler HB, Rosenblum JS, Coia LR, Moldofsky PJ, Hart WH and Broder GJ (1988) Oxygen distribution in squamous cell carcinoma metastases and its relationship in outcome of radiation therapy. *Int J Rad Oncol Biol Phys* **14**: 831–838

Giatromanolaki A, Koukourakis M, O’Byrne K, Kaklamanis L, Dicoglou C, Trichia E, Whitehouse R, Harris AL and Gatter KC (1996) Non small cell lung cancer: C-erbB-2 correlates with low angiogenesis and poor prognosis. *Anticancer Res* **16**: 3819–3825

Giatromanolaki A, Koukourakis MI, Kakolyris S, Turley H, O’Byrne K, Scott PAE, Pezzella F, Georgoulias V, Harris AL and Gatter KC (1998) Vascular endothelial growth factor, wild-type p53 and angiogenesis in early operable non-small cell lung cancer. *Clin Cancer Res* **4**: 3017–3024

Giatromanolaki A, Sivridis E, Koukourakis MI, Georgoulias V, Gatter KC and Harris AL (1999) Intratumoral angiogenesis: a new prognostic indicator for stage I endometrial adenocarcinomas? *Oncol Res* **11**: 205–212

Giatromanolaki A, Koukourakis MI, Sivridis E, O’Byrne K, Cox G, Thorpe PE, Gatter KC and Harris AL (2000) co-expression of MUC1 glycoprotein with multiple angiogenic factors in non-small cell lung cancer suggets co-activation of angiogenic and migratory pathways. *Clin Cancer Res* **6**: 1917–1921

Goldberg MA, Dunning SP and Bunn HF (1998) Regulation of the erythropoietin gene: evidence that oxygen sensor is a heme protein. *Science* 1412–1415

Gopfert T, Gess B, Eckardt KU and Kurtz A (1996) Hypoxia signalling in the control of erythropoietin gene expression in rat hepatocytes. *J Cell Physiol* **168**: 354–361

Griffiths L, Dachs GU, Bicknell R, Harris AL and Stratford IJ (1997) The influence of oxygen-tension and pH on the expression of platelet-derived endothelial cell growth factor thymidine phosphorylase in human breast-tumor cells grown in vitro and in vivo. *Cancer Res* **57**: 570–572

Gu YZ, Moran SM, Hogenesch JB, Wartman L and Bradfield CA (1998) Molecular characterization and chromosomal localization of a third α-class hypoxia inducible factor subunit, HIF3alpha. *Gene Expr* **7**: 205–213

Huang LE, Arany Z, Livingston DM and Bunn HF (1996) Activation of Hypoxia-inducible transcription factor depends primarily upon redox-sensitive stabilization of its a subunit. *J Biol Chem* **271**: 32253–32259

Huang LE, Gu J, Scheau M and Bunn F (1998) Reulation of hypoxia-inducible factor 1α is mediated by an $O_2$-dependent degradation domain via the ubiquitine-proteasome pathway. *Proc Natl Acad Sci USA* **95**: 7987–7992

Jiang BH, Agani F, Passaniti A and Semenza GL (1997) V-SRC induces expression of hypoxia inducible factor 1 (HIF-1) and transcription of genes encoding vascular endothelial growth factor and enolase-1: involvement of HIF-1 in tumor progression. *Cancer Res* **57**: 5328–5335

Koukourakis MI, Giatromanolaki A, O’Byrne K, Comley M, Whitehouse R, Talbot DC, Gatter KC and Harris AL (1997a) Platelet-derived endothelial cell growth factor expression correlates with tumor angiogenesis and prognosis in non-small cell lung cancer. *Br J Cancer* **4**: 477–481

Koukourakis MI, Giatromanolaki A, O’Byrne K, Whitehouse R, Talbot DC, Gatter KC and Harris AL (1997b) Potential role of bcl-2 as a suppressor of tumour angiogenesis in non small cell lung cancer. *Int J Cancer* **74**: 565–570

Koukourakis M, Giatromanolaki A, Kakolyris S, O’Byrne K, Apostolikas T, Skarlatos J, Gatter KC and Harris AL (1998) Different patterns of stromal and cancer cell thymidine phosphorylase reactivity in non small cell lung cancer. Impact on neoangiogenesis and survival. *British J Cancer* **77**: 1696–1703

Koukourakis MI, Giatromanolaki A, O’Byrne KJ, Cox J, Krammer B, Gatter KC and Harris AL (2000a) bcl-2 and c-erbB-2 proteins are involved in the regulation of VEGF and of Thymidine phosphorylase angiogenic activity in non-small cell lung cancer. *Clin Exp Metast* **17**: 545–554

Koukourakis MI, Giatromanolaki A, Thorpe PE, Brekken RA, Sivridis E, Kakolyris S, Georgoulias V, Gatter KC and Harris AL (2000b) Prognostic and pathologic comparison of VEGF/KDR activated microvessel density (aMVD) vs. CD31 standard MVD (sMVD) in non-small cell lung cancer. *Cancer Res* **60**: 3088–3095

Maxwell PH, Dachs GU, Gleadle JM, Nicholls LG, Harris AL, Stratford IJ, Hankinson O, Pugh CW and Ratclife PJ (1997) Hypoxia-inducible factor 1 modulates gene expression in solid tumors and influences both angiogenesis and tumor growth. *Proc Natl Acad Sci USA* **94**: 8104–8109

Maxwell PH, Wiesener MS, Chang GW, Clifford SC, Vaux EC, Cockman ME, Wykoff CC, Pugh CW, Maher ER and Ratcliffe PJ (1999) The tumour suppressor protein VHL targets hypoxia-inducible factors for oxygen-dependent proteolysis. *Nature* **399**: 271–275

Mazure NM, Chen EY, Laderoute KR and Giaccia AJ (1999) Induction of vascular endothelial growth factor by hypoxia is modulated by a phosphatidylinositol 3-kinase/Akt signaling pathway in Haras-transformed cells through a hypoxia inducible factor-1 transcriptional element. *Blood* **90**: 3322–3331

Norris ML and Millhorn DE (1995) Hypoxia-induced protein binding to O2-responsive sequences on the tyrosine hydroxylase gene. *J Biol Chem* **270**: 23774–23779

O’Toole EA, Marinkovich MP, Peavey CL, Amieva MR, Furthmayr H, Mustoe TA and Woodley DT (1997) Hypoxia increases human keratinocyte motility on connective tissue. *J Clin Invest* **100**: 2881–2891

Palmer LA, Semenza GL, Stoler MH and Johns RA (1998) Hypoxia induces type II NOS gene expression in pulmonary artery endothelial cells via HIF-1. *Am J Physiol* **274**: 212–219

Semenza GL and Wang GL (1992) A nuclear factor induced by hypoxia via de novo protein synthesis binds to the human erythropoietin gene enhancer at a site required for transcriptional activation. *Mol Cell Biol* **12**: 5447–5454

Semenza GL, Nejfelt MK, Chi SM and Antonarakis SE (1991) Hypoxia-inducible nuclear factors bind to an enhancer element located 3′ to the human erythropoietin gene. *Proc Natl Acad Sci USA* **88**: 5680–5684

Takagi H, King GL and Aiello LP (1998) Hypoxia upregulates glucose transport activity through an adenosine-mediated increase of GLUT1 expression in retinal capillary endothelial cells. *Diabetes* **47**: 1480–1488

Talks KL, Turley H, Gatter KC, Maxwell PH, Pugh CW, Ratcliffe PJ and Harris AL (2000) The expression and distribution of the hypoxia inducible factors HIF-1á and HIF-2a in normal human tissues, cancers and tumor associated macrophages. *Am J Pathol* **157**: 2411–2421

Tian H, McKnight L and Russel DW (1997) Endothelial PAS domain protein 1 (EPAS1), a transcription factor selectively expressed in endothelial cells. *Genes Dev* **11**: 72–82

Tungekar MF, Gatter KC, Dunnill MS and Mason DY (1991) Ki-67 immunostaining and survival in operable lung cancer. *Histopathology* **19**: 545–550

Volm M and Koomagi R (2001) Hypoxia-inducible factor (HIF-1) and its relationship to apoptosis and proliferation in lung cancer. *Anticancer Res* **20**: 1527–1533

Wiesener MS, Turley H, Allen WE, Willam C, Eckardt KU, Talks KL, Wood SM, Gatter KC, Harris AL, Pugh CW, Ratcliffe PJ and Maxwell PH (1998) Induction of endothelial PAS domain protein-1 by hypoxia: characterization and comparison with hypoxia-inducible factor-1α. *Blood* **92**: 2260–2268

Zagzag D, Zhong H, Scalzitti JM, Laughner E, Simons JW and Semenza GI (2000) Expression of hypoxia-inducible factor 1alpha in brain tumours: association with angiogenesis, invasion, and progression. *Cancer* **88**: 2606–2628

© 2001 Cancer Research Campaign

Zhong H, De Marzo AM, Laughner E, Lim M, Hilton DA, Zagzag D, Buechler P, Isaacs WB, Semenza GL and Simons JW (1999) Overexpression of Hypoxia-inducible factor 1α in common human cancers and their metastases. *Cancer Res* **59**: 5830–5835

Zundel W, Schindler C, Haas-Kogan D, Koong A, Kaper F, Chen E, Gottschalk AR, Ryan HE, Johnson RS, Jefferson AB, Stokoe D and Giaccia AJ (2000) Loss of PTEN facilitates HIF-1-mediated gene expression. *Genes Dev* **14**: 391–396

 *© 2001 Cancer Research Campaign*
